# Associations of HER2 Mutation With Immune-Related Features and Immunotherapy Outcomes in Solid Tumors

**DOI:** 10.3389/fimmu.2022.799988

**Published:** 2022-02-23

**Authors:** Deqiang Wang, Xiaofeng Chen, Yian Du, Xiaoqin Li, Leqian Ying, Yi Lu, Bo Shen, Xuan Gao, Xin Yi, Xuefeng Xia, Xinbing Sui, Yongqian Shu

**Affiliations:** ^1^ Department of Medical Oncology, Affiliated Hospital of Jiangsu University, Zhenjiang, China; ^2^ Department of Medical Oncology, Jiangsu Province Hospital, Nanjing, China; ^3^ The Cancer Hospital of the University of Chinese Academy of Sciences (Zhejiang Cancer Hospital), Institute of Basic Medicine and Cancer, Chinese Academy of Sciences, Hangzhou, China; ^4^ Department of Medical Oncology, The Affiliated Cancer Hospital of Nanjing Medical University, Nanjing, China; ^5^ State Key Laboratory of Microbial Resources, Institute of Microbiology, Chinese Academy of Sciences, Beijing, China; ^6^ Shenzhen Clinical Laboratory, GenePlus, Shenzhen, China; ^7^ Beijing Institute, GenePlus, Beijing, China; ^8^ School of Pharmacy, Hangzhou Normal University, Hangzhou, China

**Keywords:** immunotherapy, immune checkpoint inhibitors, HER2, tumor microenvironment, immunity

## Abstract

**Background:**

HER2 is one of the most extensively studied oncogenes in solid tumors. However, the association between tumor microenvironment (TME) and HER2 mutation remains elusive, and there are no specific therapies for HER2-mutated tumors. Immune checkpoint inhibitors (ICIs) have been approved for some tumor subgroups that lack targeted therapies, while their effects are still unclear in HER2-mutated tumors. We examined whether HER2 mutation impacts treatment outcomes of ICIs in solid tumors *via* its association with anticancer immunity.

**Methods:**

Multi-omics data of solid tumors from The Cancer Genome Atlas (TCGA), the Asian Cancer Research Group and the Affiliated Hospital of Jiangsu University were used to analyze the association between HER2 mutations and tumor features. Data of patients with multiple microsatellite-stable solid tumors, who were treated by ICIs including antibodies against programmed cell death-1 (PD-1), programmed cell death ligand-1 (PD-L1), or cytotoxic T lymphocyte-associated protein 4 (CTLA-4) in eight studies, were collected to investigate the effects of HER2 mutations on immunotherapy outcomes.

**Results:**

The mutation rate of HER2 varied in solid tumors of TCGA, with an overall incidence of 3.13%, ranged from 0.39% to 12.2%. Concurrent HER2 mutations and amplifications were rare (0.26%). HER2 mutation was not associated with HER2 protein expression but was positively associated with microsatellite instability, tumor mutation and neoantigen burdens, infiltrating antitumor immune cells, and signal activities of antitumor immunity. Of 321 ICI-treated patients, 18 carried HER2 mutations (5.6%) and showed improved objective response rates compared with those with HER2 wild-type (44.4% vs. 25.7%, p=0.081), especially in the anti-PD-1/anti-PD-L1 subgroup (62.5% vs. 28.4%, p=0.04). Heterogeneity was observed among tumor types. Patients with HER2 mutations also had superior overall survival than those with HER2 wild-type (HR=0.47, 95%CI: 0.23-0.97, p=0.04), especially in the presence of co-mutations in ABCA1 (HR = 0.23, 95% CI: 0.07-0.73, p=0.013), CELSR1 (HR = 0.24, 95% CI: 0.08-0.77, p=0.016), LRP2 (HR = 0.24, 95% CI: 0.07-0.74, p=0.014), or PKHD1L1 (HR = 0.2, 95% CI: 0.05-0.8, p=0.023).

**Conclusions:**

HER2 mutations may improve the TME to favor immunotherapy. A prospective basket trial is needed to further investigate the impacts of HER2 mutations on immunotherapy outcomes in solid tumors.

## Background

Human epidermal growth factor 2 (HER2), encoded by the ERBB2 gene, is one of the most extensively studied oncogenes in solid tumors. HER2 alterations, with three principal mechanisms (protein overexpression, gene amplification, and gene mutation), have crucial roles in cancer progression and are important therapeutic targets (1).

For HER2 overexpression or amplification, known as HER2-positive, HER2-targeted therapies have been demonstrated to improve survival in patients with breast cancer in the adjuvant, neoadjuvant, and salvage settings, as well as in patients with advanced stomach adenocarcinoma (STAD) (1). HER2-targeted therapies are also adopted in other cancers (such as non-small cell lung cancer [NSCLC] and colorectal cancer) when HER2-positive was identified, although they are still not approved for those patients due to inadequate evidence (1). More recently, novel HER2-targeted drugs (i.e., antibody-drug conjugates [ADCs]), such as ado-trastuzumab emtansine (T-DM1) and trastuzumab deruxtecan (T-DXd, also known as DS8201a), have demonstrated promising antitumor activity in multiple advanced solid tumors that are HER2-positive (2–5).

Unlike HER2 overexpression or amplification, most HER2 mutations tend to be resistant to HER2-targeted therapies, even in breast cancer (6). Depending on the mutation type and concomitant genomic aberrations, other anti-HER2 drugs, such as afatinib, a pan-HER inhibitor, have moderate efficacy in NSCLC patients with HER2 mutations (7, 8). ADC seems to be more effective than traditional anti-HER2 drugs in HER2-mutated tumors, and objective response rates (ORRs) of 44% and 72.7% have been reported for T-DM1 and T-DXd, respectively, in HER2-mutated NSCLC (3, 9). However, these results are based on subgroup analyses with few patients in phase I/II trials and are thus inconclusive. Consequently, to date, no targeted therapies have been approved for treating HER2-mutated tumors.

Immunotherapy with immune checkpoint inhibitors (ICIs), including antibodies against programmed cell death-1 (PD-1), programmed cell death ligand-1 (PD-L1), and cytotoxic T lymphocyte-associated protein 4 (CTLA-4), has achieved success in some tumor subgroups that lack targeted therapies, such as NSCLC without typical driver mutations (10). Thus, immunotherapy has been investigated in HER2-mutated subgroups, especially in NSCLC. For example, a retrospective study reported that the ORR, median progression-free survival (PFS), and overall survival (OS) rates at 1 year were respectively 52%, 6 months, and 88% in 27 patients with advanced NSCLC who carried HER2 mutations and received first-line ICIs in combination with chemotherapy (11). For second/third-line treatment in HER2-mutated NSCLC, single ICIs are usually used; a retrospective study observed an ORR of 29% and a median PFS of 3.6 months in 14 patients (12), and another real-world study showed an ORR of 27.3% and a median OS of 20.4 months in 23 patients (13). These results are similar to those observed in unselected NSCLC patients. However, two other retrospective studies with a total of 10 NSCLC patients with HER2 mutations reported an ORR of zero (14, 15). Therefore, ICI efficacy remains unclear in HER2-mutated NSCLC and other tumors.

Accordingly, the present pan-cancer study aimed to investigate the associations between HER2 mutation and immune-related features or immunotherapy outcomes. Our findings suggest that HER2 mutation may improve the tumor microenvironment (TME) to favor immunotherapy, despite heterogeneity among various types of tumors.

## Materials and Methods

### Pan-Cancer Cohort

The pan-cancer cohort in The Cancer Genome Atlas (TCGA) Pan-Cancer project was used as the discovery set, which enables robust cross-tumor-type analyses. Multidimensional data including somatic mutations, copy number variants, mRNA expression profiles, microsatellite instability (MSI) scores reported by MSIsensor (threshold of MSI >10), and protein expression measured by reverse-phase protein array (RPPA) were downloaded from the cBioPortal database (http://www.cbioportal.org/). Tumor proliferation scores, interferon-gamma response scores, abundances of tumor-infiltrating immune cells calculated based on transcriptome data, and tumor neoantigen burden (TNB) were obtained from the supplementary materials in the literature about the immune landscape of TCGA pan-cancer (16).

### Independent STAD Cohort

STAD, a globally common cancer, is well known for its wide heterogeneity, which causes dissatisfied treatment efficacy and high mortality (17, 18). STAD may be an appropriate tumor type to examine the effect of a pan-cancer biomarker because classical pan-cancer biomarkers including MSI, tumor mutation burden (TMB), and PD-L1 expression all contribute to the prediction of immunotherapy outcomes in STAD (19). In addition, HER2 alterations and other indices evaluated in this study are essential in STAD. Thus, STAD cohorts from the Affiliated Hospital of Jiangsu University (AHJU) (20) and the Asian Cancer Research Group (ACRG) (18) were used as validation sets. For the AHJU cohort, written informed consent was obtained from all patients, and the research protocol was approved by the Ethics Committee of AHJU. Genome data of AHJU were stored in the dataset-HRA001647 in the Genome Sequence Archive for Human. For ACRG, transcriptome data were downloaded from the NCBI Gene Expression Omnibus (GSE62254), and genomic data were obtained from the supplementary materials in the published literature (18).

### Immunotherapy Cohort

Published immunotherapy cohorts were screened based on genomic data, detailed classification of HER2 variants, and available data on response evaluation and survival. Finally, data of ICI-treated patients with multiple microsatellite-stable (MSS) solid tumors from eight studies were collected to investigate the effects of HER2 mutations on immunotherapy outcomes (21–28).

### Whole Exome Sequencing (WES)

WES was performed in the AHJU STAD cohort, as previously described (20). After extraction of genomic DNA, the whole genome library was prepared using the KAPA Hyper Prep Kit (KAPA Biosystems), and exomes were captured using the Illumina Rapid Capture Extended Exome Kit (Illumina Inc.). The Illumina HiSeq4000 NGS platform (Illumina, USA) was used to sequence enriched libraries as paired 150-bp reads. The data processing has been described in detail elsewhere (29).

### HER2 Mutation/Amplification and TMB

This study only considered somatic nonsynonymous variations (SNVs) in HER2, including missense, nonsense, splice-site, and frameshift mutations (30). The total SNV counts in the coding regions were defined as TMB. The putative copy number alterations of HER2 ≥ 2 were evaluated for amplification.

### MSI Assay

In the AHJU STAD cohort, MSI status was determined by single fluorescent multiplex PCR based on five well-known mononucleotide repeats (18). MSI was defined as allelic size variations in more than one microsatellite in the samples.

### Multiple-Immunofluorescence (mIF) Staining

As previously described, mIF staining was conducted using a PANO 7-plex IHC kit (Panovue, Beijing, China) (31). Antibodies used included anti-panCK (CST4545, Cell Signaling Technology, Danvers, MA, USA), anti-CD8 (CST70306), anti-CD56 (CST3576), anti-CD68 (BX50031, Biolynx, China), and anti-HLA-DR (ab92511, Abcam, UK). The stained slides were scanned, and a single stacked image was constructed using the Mantra System (PerkinElmer, Waltham, MA, US). Furthermore, images of the sections were reconstructed based on a spectral library of multispectral unmixing. Finally, various cells were counted using the InForm image analysis software (PerkinElmer).

### Statistical Analysis

The proportional composition of two variables was compared using the Chi-square or Fisher’s exact tests. The comparison of means between the two datasets was performed using Mann-Whitney *U* tests. Survival analysis was conducted using the Kaplan–Meier method and log-rank test. The Cox proportional hazards model was used to evaluate the effects of HER2 mutations on survival, and hazard ratios (HRs) and their 95% confidence intervals (CIs) were calculated. All statistical analyses were performed using SPSS (version 19.0, Chicago, IL, USA), R (version 3.6.1), and R Bioconductor packages. All tests were two-sided, and statistical significance was set at p <0.05.

## Results

### Prevalence of HER2 Mutation and Its Association With HER2 Amplification

**Figure 1:**
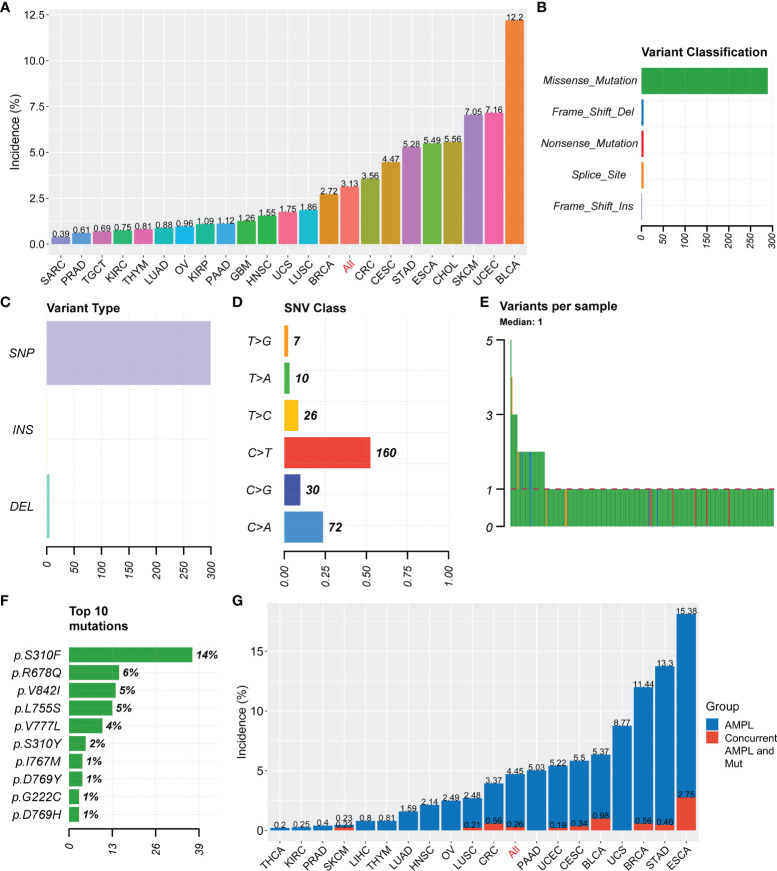
Summary of HER2 mutation in solid tumors. **(A)** Incidences of HER2 mutation in solid tumors from the TCGA Pan-Cancer project. **(B–F)** Variant classification **(B)**, variant type **(C)**, SNV class **(D)**, variants per sample **(E)**, and the top 10 most common mutations **(F)** for HER2 mutation. **(G)** Incidences of HER2 amplification (AMPL) and concurrent HER2 mutation (Mut) and amplification in solid tumors. SNV, somatic nonsynonymous variation; TCGA, The Cancer Genome Atlas; SARC, Sarcoma; PRAD, Prostate adenocarcinoma; TGCT, Testicular Germ Cell Tumors; KIRC, Kidney renal clear cell carcinoma; THYM, Thymoma; LUAD, Lung adenocarcinoma; OV, Ovarian serous cystadenocarcinoma; KIRP, Kidney renal papillary cell carcinoma; PAAD, Pancreatic adenocarcinoma; GBM, Glioblastoma multiforme; HNSC, Head and Neck squamous cell carcinoma; UCS, Uterine Carcinosarcoma; LUSC, Lung squamous cell carcinoma; BRCA, Breast invasive carcinoma; CRC, colorectal cancer; CESC, Cervical squamous cell carcinoma and endocervical adenocarcinoma; STAD, Stomach adenocarcinoma; ESCA, Esophageal carcinoma; CHOL, Cholangio carcinoma; SKCM, Skin Cutaneous Melanoma; UCEC, Uterine Corpus Endometrial Carcinoma; BLCA, Bladder Urothelial Carcinoma; THCA, Thyroid carcinoma.

HER2 mutations were observed in 22 tumor types from the TCGA pan-cancer project. The mutational frequency of HER2 was 3.13% (261 of 8328 patients; range: 0.39%-12.2%) in various cancer types (**Figure 1A**). Most HER mutations were missense single nucleotide polymorphisms, with a DNA base change from cytosine to thymine (**Figures 1B–D**). Patients usually carried only one HER2 mutation, in which p.S310F was the most common substitution (14%; **Figures 1E, F**). According to patient characteristics, the HER2 mutation rate was significantly higher in non-squamous cell carcinomas than in squamous cell carcinomas (3.2% vs. 1.8%, p = 0.007), as well as in histological grade III/IV than in I/II carcinomas (5.1% vs. 3.2%,p = 0.006) (**Table 1**). Moreover, the co-occurrence of HER2 mutation and amplification was rare (only in 10 tumor types), with a proportion of 0.26% (24 of 9184 patients) in the overall cohort consisting of tumor types carrying HER2 mutation or amplification (**Figure 1G**). These results indicate that HER2 mutations and amplification may be mutually exclusive.

**Table 1:** Patient characteristics by HER2 mutation status in the TCGA pan-cancer cohort.

Characteristic^*^	Wild-type (%)	Mutation (%)	P value
Age (years)			
<65	4713 (97.2)	138 (2.8)	0.095
≥65	3347 (96.5)	121 (3.5)	
Sex			
Female	4206 (96.6)	149 (3.4)	0.106
Male	3782 (97.2)	109 (2.8)	
Stage			
I/II	3913 (96.5)	143 (3.5)	0.997
III/IV	2735 (96.5)	100 (3.5)	
Histological type			
Non-SCC	6324 (96.8)	206 (3.2)	0.007
SCC	1311 (98.2)	24 (1.8)	
Histological grade			
I/II	1338 (96.8)	44 (3.2)	0.006
III/IV	1879 (94.9)	102 (5.1)	

^*^Based on available data.

SCC, squamous cell carcinoma; TCGA, The Cancer Genome Atlas.

### Distinctive Tumor Features Between HER2 Mutation and Amplification

**Figure 2:**
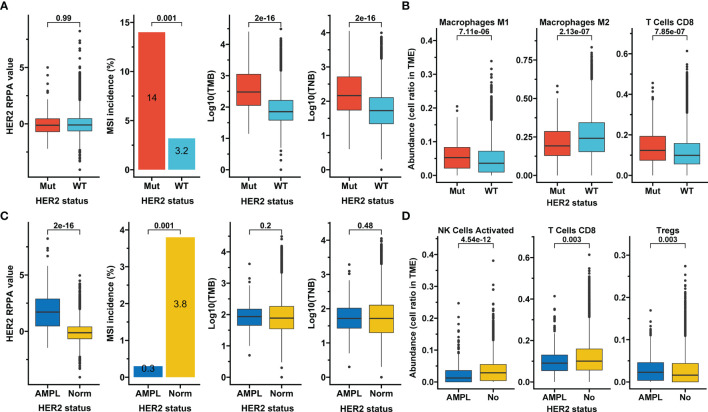
Association of HER2 alterations with tumor features. **(A, B)** Association of HER2 mutation with HER2 protein expression, microsatellite instability (MSI), tumor mutation burden (TMB), tumor neoantigen burden (TNB) **(A)**, and selected infiltrating immune cells whose aundances were calculated based on transcriptome data and downloaded from the immune landscape of TCGA **(B)**. **(C, D)** The same analysis items as in A for HER2 amplification. RPPA, reverse-phase protein array; Mut, mutation; WT, wild-type; AMPL, amplification; Norm, normal; TME, tumor microenvironment; Tregs, regulatory T cells; TCGA, The Cancer Genome Atlas.

In the overall TCGA Pan-Cancer cohort, although the HER2 protein expression measured by RPPA was not altered, significantly higher MSI incidence (14% vs. 3.2%), TMB, and TNB were observed in samples with HER2 mutations than in those with HER2 wild-type (p<0.05; **Figure 2A**). HER2 mutation subgroup also had higher abundances of tumor-infiltrating M1 macrophages and CD8+ T cells but a lower abundance of M2 macrophages (p<0.05; **Figure 2B**). In contrast, HER2 amplification presented a higher HER2 protein expression but a lower MSI incidence (0.3% vs. 3.8%, p=0.001) than normal HER2 control, and was irrelevant to TMB and TNB (**Figure 2C**). Moreover, HER2 amplification subgroup had a higher abundance of tumor-infiltrating regulatory T cells but lower abundances of activated NK cells and CD8+ T cells (p<0.05; **Figure 2D**). These results suggest that HER2 mutation and amplification may have different effects on antitumor immunity and immunotherapy efficacy.

### Heterogeneity of HER2-Associated Features According to Tumor Types

HER2 mutation was not associated with HER2 protein expression in all tumor types except in head and neck squamous cell carcinomas (HNSC) (**Supplementary Figure S1**). HER2 mutation generally accompanied a higher TMB than HER2 wild-type in various tumors, except kidney renal papillary cell carcinoma, lung squamous cell carcinoma (LUSC), and uterine carcinosarcoma (UCS) (**Supplementary Figure S2**). HER2 mutation also accompanied a higher TNB than HER2 wild-type in most tumor types, except LUSC, sarcoma, cutaneous skin melanoma, and UCS (**Supplementary Figure S3**).

For HER2 amplification, a greater HER2 protein expression than normal HER2 control was observed in almost all tumor types, except kidney renal clear cell carcinoma (**Supplementary Figure S4**). HER2 amplification was positively associated with TMB in invasive breast carcinoma, esophageal carcinoma, and pancreatic adenocarcinoma (PAAD), but was negatively associated with TMB in uterine corpus endometrial carcinoma (UCEC) (p<0.05; **Supplementary Figure S5**). Moreover, HER2 amplification was positively associated with TNB in PAAD and STAD, but was negatively associated with TNB in UCEC (p<0.05; **Supplementary Figure S6**). In addition, in four tumor types with an MSI incidence greater than 1%, HER2 mutation was positively associated with MSI incidence in all these tumors (p<0.05), while HER2 amplification had an opposite effect (**Supplementary Figure S7** and **Tables S1**, **S2**).

These findings revealed that the identical HER2 alteration type had similar effects on most tumor types, but heterogeneous influences were also observed in some tumor types. However, few cases of HER2 changes and incomplete TNB data in many tumor types had limited our analyses.

### Validation of HER2 Mutation’s Effect on Immune-Related Features in Independent STAD Cohorts

**Figure 3:**
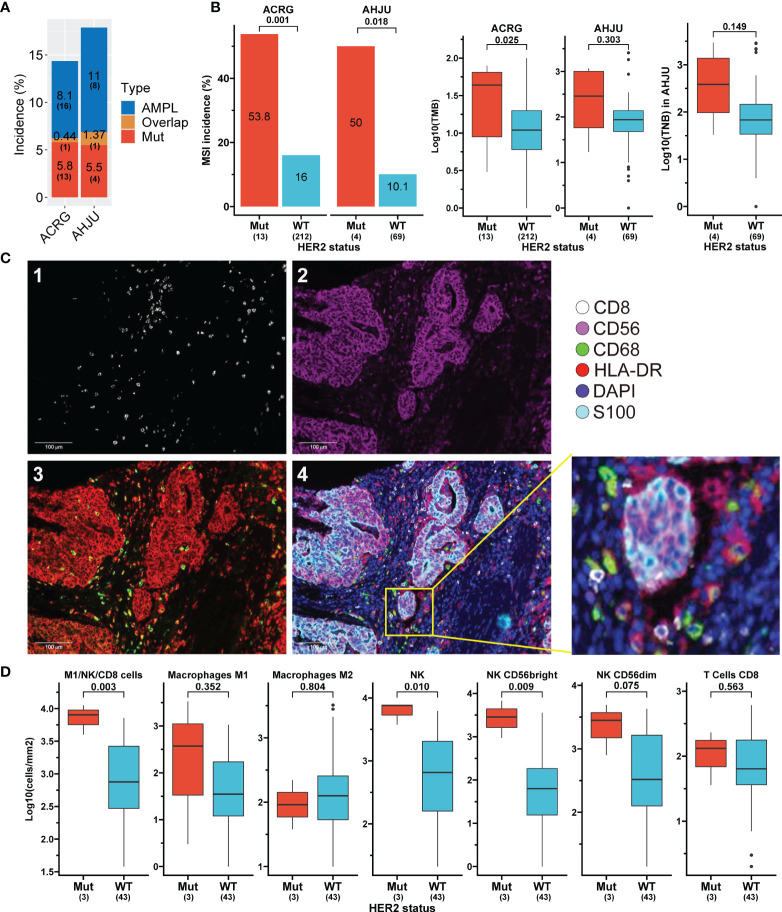
Association of HER2 mutation with tumor features validated in independent stomach adenocarcinoma (STAD) cohorts. **(A)** Incidences of HER2 alterations in ACRG and AHJU. **(B)** Association of HER2 mutation with microsatellite instability (MSI), tumor mutation burden (TMB), and tumor neoantigen burden (TNB). **(C)** Multiple-immunofluorescence staining of surface biomarkers of immune cells in STAD tissues. 1: CD8 staining; 2: CD56 staining; 3: CD68 (green) and HLA-DR (red) staining; 4: the reconstructed image for all surface biomarkers. **(D)** The density of infiltrating immune cells in tumor parenchyma stratified by HER2 mutation status. ACRG, Asian Cancer Research Group; AHJU, Affiliated Hospital of Jiangsu University; AMPL, amplification; Mut, mutation; WT, wild-type.

The aforementioned findings indicate a favorable influence of HER2 mutation on anticancer immunity. These findings were further verified in two independent STAD cohorts, the ACRG and AHJU. The HER2 mutation incidence was 5.8% (13 of 225 patients) and 5.5% (4 of 73 patients) in the ACRG and AHJU groups, respectively (**Figure 3A**). The co-occurrence of HER2 mutation and amplification was still rare, with proportions of 0.44% and 1.37% in ACRG and AHJU, respectively. These data are similar to those of TCGA STAD (**Figures 1A, G**). HER2 mutation was positively associated with MSI incidence, TMB, and TNB, although the statistical significance of some results was limited by the small sample size of AHJU (**Figure 3B**).

We further validated the impact of HER2 mutation on immune infiltration by mIF staining in 46 AHJU samples (**Figure 3C**). HER2 mutation was positively associated with the infiltration of total NK cells and NK CD56bright subtype cells in tumor parenchyma (p = 0.01 and 0.009, respectively) and seemed to be positively associated with the infiltration of M1 macrophages, CD8+ T cells, and NK CD56dim subtype cells. Moreover, HER2 mutation was positively associated with the infiltration of overall anticancer cells, including M1, NK, and CD8+ T cells (p = 0.003) (**Figure 3D**).

### HER2 Mutation Has Immune-Associated Transcriptome Features

**Figure 4:**
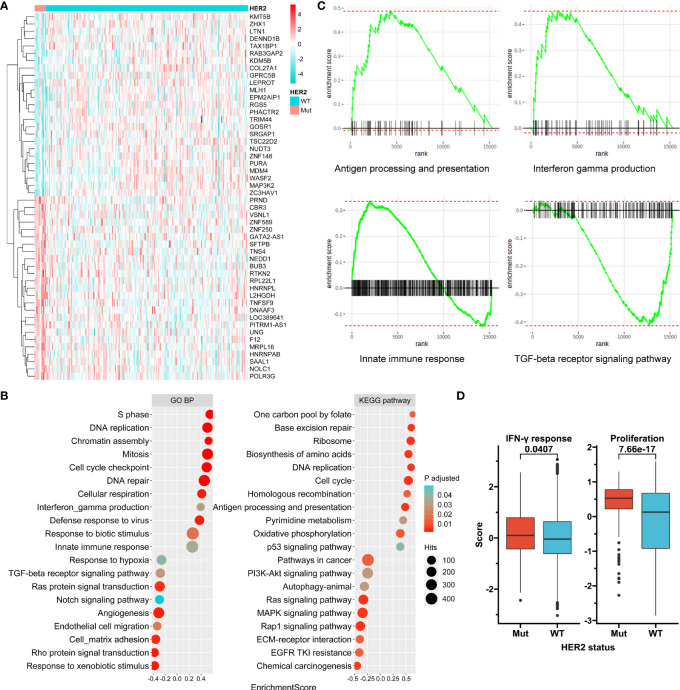
Transcriptome features associated with HER2 mutation. **(A)** A clustered heat map for top 40 genes differentially expressed between HER2 mutation (Mut) and wild-type (WT) subgroups in ACRG. **(B)** Selected enriched signaling pathways based on Kyoto Encyclopedia of Genes and Genomes (KEGG) and biological process (BP) terms of Gene Ontology (GO) in gene set enrichment analysis (GSEA). **(C)** Enrichment plots for selected immune-associated signaling or functions. **(D)** Tumor proliferation and interferon-gamma response scores according to HER2 mutation status in the TCGA Pan-Cancer cohort. ACRG, Asian Cancer Research Group; TCGA, The Cancer Genome Atlas.

Transcriptome data from the ACRG were used to compare gene expression between HER2 mutation and HER2 wide-type subgroups (**Figure 4A**). Gene set enrichment analysis revealed that genes involved in proliferation-associated and immune-associated signaling or functions were significantly enriched in the HER2 mutation subgroup (**Figure 4B**). Specifically, antigen processing and presentation, interferon-gamma production, and the innate immune response seemed to be activated by HER2 mutation, while the transforming growth factor-beta receptor signaling pathway, the driver of immune exclusion, might be inhibited (**Figure 4C**). Furthermore, we showed that both tumor proliferation and interferon-gamma response scores were significantly higher in the HER2 mutation subgroup than in the HER2 wide-type subgroup in the TCGA Pan-Cancer cohort (**Figure 4D**).

### HER2 Mutation Impacts Immunotherapy Outcomes

Due to the low incidence of HER2 mutation, data of ICI-treated patients with solid tumors were pooled from eight studies, and we found that all these patients were MSS. A total of 321 patients with five tumor types were included in our study (**Table S3**). Of these, 145 patients received anti-CTLA-4 therapy, 166 received anti-PD-1/anti-PD-L1 therapy, and 10 received a combination of anti-CTLA-4 and anti-PD-1/anti-PD-L1 therapies. HER2 mutations were detected in 18 patients (5.6%).

**Figure 5:**
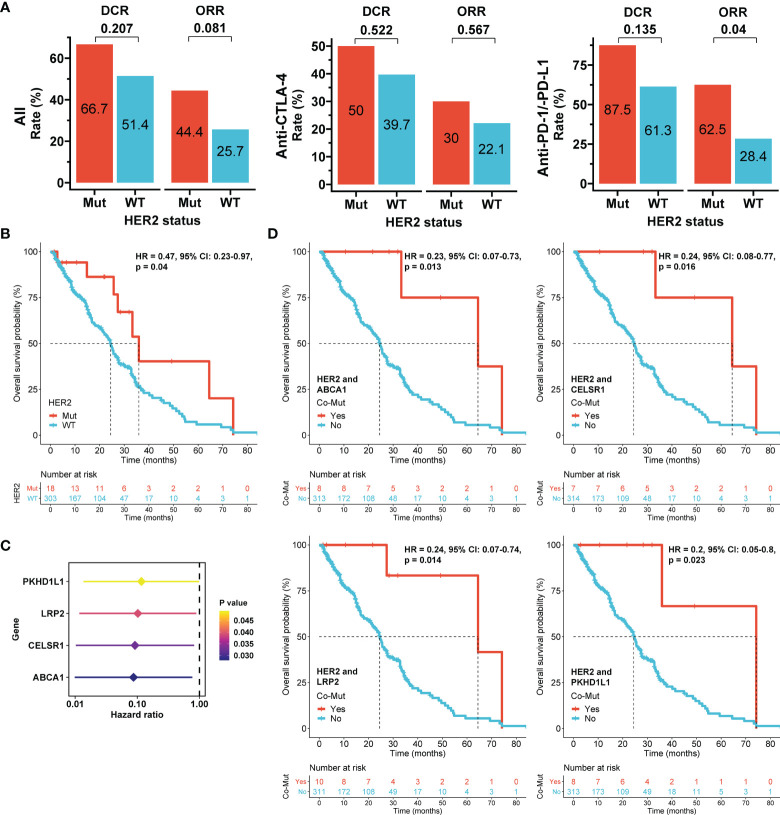
Association of HER2 mutation with immunotherapy outcomes in the pooled immunotherapy cohort. **(A)** Objective response rate (ORR) and disease control rate (DCR) according to HER2 mutation status and stratified by immune checkpoint inhibitors. Differences between subgroups were compared using the Chi-square or Fisher’s exact tests. **(B)** Overall survival (OS) according to HER2 mutation status. **(C)** Hazard ratios of genes with mutations that were significantly associated with OS in univariate Cox models in HER2-mutated tumors. **(D)** OS according to co-mutations (Co-Mut). Mut: mutation; WT: wild-type. The Cox proportional hazards model was used to evaluate the effects of HER2 mutations or co-mutations on survival.

Patients with HER2 mutations had higher ORRs and disease control rates (DCRs) than those with wild-type HER2 in the overall cohort (44.4% vs. 25.7% and 66.7% vs. 51.4%, respectively), the anti-CTLA-4 subgroup (30% vs. 22.1% and 50% vs. 39.7%, respectively), and the anti-PD-1/anti-PD-L1 subgroup (62.5% vs. 28.4% and 87.5% vs. 61.3%, respectively) (**Figure 5A** and **Tables 2**, **3**). No HER2 mutations were detected in patients treated with the combination of anti-CTLA-4 and anti-PD-1/anti-PD-L1, with an ORR of 30%. HER2-mutated patients seemed to be more sensitive to anti-PD-1/anti-PD-L1 therapy than anti-CTLA-4 therapy (ORR: 62.5% vs 30%, p=0.168), and the ORR significantly differed between HER2 mutation and wild-type subgroups in the anti-PD-1/anti-PD-L1 therapy (p=0.04; **Figure 5A**). However, this difference may reflect tumor type heterogeneity rather than differential drug effects, because all anti-CTLA-4 therapies were performed in melanoma and anti-PD-1/anti-PD-L1 therapies were mainly adopted in other tumor types in HER2-mutated patients (**Table S3**). When stratified by the tumor type, the ORRs in HER2 mutation and wild-type subgroups were respectively 100% (1/1) and not available for anal cancer, 50% (1/2) and 48% (12/25) for bladder cancer (BLCA), 0 (0/1) and 18.2% (2/11) for HNSC, 100% (2/2) and 27.3% (15/55) for lung cancer (p=0.027), and 33.3% (4/12) and 22.9% (47/205) for melanoma (**Table S4**).

**Table 2:** Therapy response by HER2 mutation status in the pooled immunotherapy cohort.

Therapy and response^*^	Wild-type (%)	Mutation (%)	P value
Anti-CTLA-4			
Non-response	102 (77.9)	7 (70.0)	0.567
Response	29 (22.1)	3 (30.0)	
Anti-PD-1/-PD-L1			
Non-response	111 (71.6)	3 (37.5)	0.040
Response	44 (28.4)	5 (62.5)	
All			
Non-response	220 (74.3)	10 (55.6)	0.081
Response	76 (25.7)	8 (44.4)	

^*^ Response data were available in 314 patients.

**Table 3:** Disease control by HER2 mutation status in the pooled immunotherapy cohort.

Therapy and DC^*^	Wild-type (%)	Mutation (%)	P value
Anti-CTLA-4			
PD	79 (60.3)	5 (50.0)	0.522
DC	52 (39.7)	5 (50.0)	
Anti-PD-1/-PD-L1			
PD	60 (38.7)	1 (12.5)	0.135
DC	95 (61.3)	7 (87.5)	
All			
PD	144 (48.6)	6 (33.3)	0.207
DC	152 (51.4)	12 (66.7)	

^*^ Response data were available in 314 patients.

DC, disease control (defined as response or stable disease); PD, progressed disease.

Furthermore, ICI-treated patients with HER2 mutations had a better OS than those with wild-type HER2 in the overall cohort (HR = 0.47, 95% CI: 0.23-0.97, p=0.04; **Figure 5B**). Anti-CTLA-4 therapy had longer median OS than anti-PD-1/anti-PD-L1 therapy in the HER-2 mutation subgroup (64.5 months vs 33.3 months, p=0.379; **Supplementary Figure S8**). Similar to ORR, this difference may be caused by different tumor types between patients received these two therapies. Because concurrent genetic mutations may impact immunotherapy outcomes (32), genetic mutations associated with OS were identified by Cox models in HER2-mutated patients. Mutations in ABCA1, CELSR1, LRP2, or PKHD1L1 were significantly correlated with improved prognosis (**Figure 5C**). Notably, ICI-treated patients with concurrent ABCA1 and HER2 mutations (8/321; HR = 0.23, 95% CI: 0.07-0.73, p=0.013), concurrent CELSR1 and HER2 mutations (7/321; HR = 0.24, 95% CI: 0.08-0.77, p=0.016), concurrent LRP2 and HER2 mutations (10/321; HR = 0.24, 95% CI: 0.07-0.74, p=0.014), and concurrent PKHD1L1 and HER2 mutations (8/321; HR = 0.2, 95% CI: 0.05-0.8, p=0.023) all had OS superiority compared with their corresponding controls (**Figure 5D**). The findings suggest that these co-mutations more strongly impact OS than HER2 mutation alone and may thus be used to improve patient selection in immunotherapy.

## Discussion

In this study, we revealed that HER2 mutation was associated with features of solid tumors that indicate a favorable TME, including MSI, high TMB and TNB, dynamic infiltration of antitumor immune cells, and activated immune signaling. Moreover, HER2 mutation led to more favorable outcomes of ICI treatment than wild-type HER2, especially in the presence of mutations in ABCA1, CELSR1, LRP2, or PKHD1L1. Although heterogeneity was also observed among tumor types, our findings suggest implications of HER2 mutation in ICI treatments in clinical interventions.

This study showed that HER2 mutation had a low frequency (3.1%) in the overall TCGA Pan-Cancer cohort, although several common tumor types had a mutation rate of >5%. Meanwhile, the co-occurrence of HER2 mutation and a more common variation, HER2 amplification, was rare. However, most of the samples in TCGA were from early-stage tumors, while HER2 alteration seemed to increase in advanced diseases (28). Patients with HER2 mutations or amplifications may account for approximately 10% to 20% of all cancer patients, indicating that the improvement of treatment in these patients may have an essential contribution to the overall anticancer therapy. Nevertheless, the treatment needs of tumor patients with HER2 alterations remain largely unmet, especially in patients with non-breast cancers.

Recently, immunotherapy with ICIs has provided a new opportunity to treat tumor patients with HER2 alterations. In particular, the combination of anti-PD-1 (pembrolizumab), anti-HER2 (trastuzumab), and chemotherapy has achieved a milestone of ICI efficacy in previously untreated metastatic esophagogastric cancer, which is HER2-positive: The phase II NCT02954536 trial (33) and the phase III KEYNOTE 811 trial (34) reported ORRs of 91% and 74.4%, respectively. The mechanistic basis for synergy between anti-HER2 and ICI therapies has been suggestted. Trastuzumab was found to up-regulate expressions of PD-1, PD-L1 and other genes involving immune infiltration in tumor tissues (35, 36), which could improve the efficacy of pembrolizumab. Moreover, the combination of trastuzumab and pembrolizumab enhanced T-cell anti-cancer responses by promoting T cell and dendritic cell trafficking and inducing expansion of peripheral memory T cells (37–39). However, it remains unclear whether this strategy of combination therapy is suitable for tumor patients with HER2 mutations.

The association between TME and HER2 remains elusive, although HER2 is one of the most extensively studied oncogenes in solid tumors. In this study, we showed that tumor patients with HER2 mutations may have favorable TME, while HER2 amplification might generally correlate with poor TME. Recently, a study found that a subtype of GC with the enrichment of immune signatures was characterized by down-regulated HER2 protein expression (40), which is consistent with our findings to some extent for HER2 amplification in Pan-cancer cohort. However, HER2 mutation was not associated with HER2 protein expression as shown in our study, and its mechanisms to modulate TME may attribute to functional changes which needs further investigations.

Based on the positive association between HER2 mutation and favorable TME, we further revealed that patients with HER2 mutations may also have favorable immunotherapy outcomes. Specifically, this role of HER2 mutation might not depend on MSI, although we found that HER2 mutation was positively associated with MSI incidence. In the pooled ICI-treated cohort of multiple MSS solid tumors, patients with HER2 mutations showed improved ORR, DCR, and OS compared with those with HER2 wild-type. However, ORR heterogeneity was also observed after stratification according to tumor type. In particular, no response was observed in HER2-mutated HNSC, and no ORR difference was observed between HER2 mutation and wild-type in BLCA. Interestingly, we showed that HER2 mutation was positively associated with TMB and TNB in both HNSC and BLCA of TCGA. Considering that the HER2 mutation events and the sample size (1/12 in HNSC and 2/27 in BLCA) in this cohort were small, further investigations with expanded cohorts are needed to confirm our present findings.

Co-occurring genomic mutations may determine molecular and clinical heterogeneity of oncogene-driven cancer subgroups (32). In NSCLC, KRAS-TP53 co-mutations promote an inflamed TME and may be of clinical benefit for immunotherapy (41, 42). In contrast, KRAS-STK11 co-mutations are associated with an inert or “cold” TME and may thus predict a lack of clinical benefits from ICIs (43, 44). In our study, concurrent mutations of HER2 with ABCA1, CELSR1, LRP2, or PKHD1L1 induced a more favorable prognosis of immunotherapy than HER2 mutation alone. Notably, accumulating evidence supports the impact of these genetic mutations on TME. For example, ABCA1 mediates cholesterol metabolism in immune cells, and dendritic cells with ABCA1 deficiency enhance T cell activation (45, 46); CELSR1 is involved in contact-mediated cell communication, and mutations in CELSR1 encode putative specific neoantigens in colon cancer, which promote immune infiltration (47, 48); LRP2 also participates in cholesterol metabolism, and its mutations contribute to elevated fraction of immune cells in TME by up-regulating immune-related genes (49, 50); moreover, PKHD1L1, also known as PKHDL1, is relevant to autosomal-recessive polycystic kidney disease, and its expression is up-regulated specifically in T cells following signaling activation, indicating that it may play a role in cellular immunity (51). Our findings suggest that mutations in these genes may cooperate with HER2 mutations to affect TME. Further studies are needed to verify this hypothesis.

This study has several limitations. First, the small sample size of HER2-mutated tumors led to inconclusive results, although a biologic plausibility for ICI therapies was shown in the subgroup. Second, there was significant heterogeneity among tumor types. Therefore, the impact of HER2 mutation on ICI efficacy needs to be further examined in each tumor type, especially considering the recent failure of TMB as a predictive biomarker for ICI therapies in some tumor types (52). Third, besides concurrent mutations, other factors, such as treatment lines, population heterogeneity, antitumor therapies combined with ICIs, and function features of specific HER2 mutations, may confound the ICI efficacy in HER2 mutation tumors. Finally, similar to the role of HER2 overexpression or amplification in target therapy, immunotherapy, and even chemotherapy (53), HER2 mutation may have extensive impacts on clinical practise, which needs more comprehensive studies.

In conclusion, this study indicates that HER2 mutation may favor immunotherapy, although heterogeneity exists among tumor types. Further prospective basket trials including multiple tumor types with sufficient sample sizes are needed to investigate immunotherapy outcomes stratified by HER2 mutation status.

## Data Availability Statement

The datasets presented in this study can be found in online repositories. The names of the repository/repositories and accession number(s) can be found in the article/**Supplementary Material**.

## Ethics Statement

The studies involving human participants were reviewed and approved by ethics committee of the Affiliated Hospital of Jiangsu University (AHJU). The patients/participants provided their written informed consent to participate in this study.

## Author Contributions

DW, XC, YD, XS, and YS designed the study. DW, XC, and YD wrote the first draft of the manuscript. DW, XC, YD, XL, LY, YL, and BS acquired data. DW, XC, YD, LY, BS, XG, XY, XS, and YS analyzed the data. DW, XX, XS, and YS interpret the data. XL, LY, XG, XS, and YS revised the manuscript. All authors contributed to the article and approved the submitted version.

## Funding

The study was funded by China Postdoctoral Science Foundation (2021M693272), Science and Technology Planning Social Development Project of Zhenjiang City (SH2021068), Innovation Funds From Chinese Society Of Clinical Oncology Youth Committee (Y-Young2021-0107), Project of Young Medical Talents in Jiangsu Province (QNRC2016829), and 5123 Scholar Program of the Affiliated Hospital of Jiangsu University (51232017301).

## Conflict of Interest

XG is the employee of GenePlus-Shenzhen Clinical Laboratory. XY and XX are the employees of Geneplus-Beijing Institute.

The remaining authors declare that the research was conducted in the absence of any commercial or financial relationships that could be construed as a potential conflict of interest.

## Publisher’s Note

All claims expressed in this article are solely those of the authors and do not necessarily represent those of their affiliated organizations, or those of the publisher, the editors and the reviewers. Any product that may be evaluated in this article, or claim that may be made by its manufacturer, is not guaranteed or endorsed by the publisher.

## Supplementary Material

Supplementary Figure S1 Impact of HER2 mutation on HER2 protein expression by tumor types. RPPA, reverse-phase protein array; Mut, mutation; WT, wild-type.

Supplementary Figure S2 Impact of HER2 mutation on tumor mutation burden (TMB) by tumor types. Mut, mutation; WT, wild-type.

Supplementary Figure S3 Impact of HER2 mutation on tumor neoantigen burden (TNB) by tumor types; Mut, mutation; WT, wild-type.

Supplementary Figure S4 Impact of HER2 amplification on HER2 protein expression by tumor types. RPPA, reverse-phase protein array; AMPL, amplification; Norm, normal.

Supplementary Figure S5 Impact of HER2 amplification on tumor mutation burden (TMB) by tumor types. AMPL, amplification; Norm, normal.

Supplementary Figure S6 Impact of HER2 amplification on tumor neoantigen burden (TNB) by tumor types. AMPL, amplification; Norm, normal.

Supplementary Figure S7 Impact of HER2 alterations on microsatellite instability (MSI) by tumor types. **(A, B)** HER2 mutation **(A)** and amplification **(B)** impact MSI in 4 tumor types with a MSI incidence larger than 1%. Mut, mutation; WT, wild-type; AMPL, amplification; Norm, normal copy number. The MSI composition between subgroups was compared using the Chi-square or Fisher’s exact tests.

Supplementary Figure S8 Overall survival stratified by types of immune checkpoint inhibitors in the pooled immunotherapy cohort.
