# Supplementary figures and images for: Associations of HER2 Mutation With Immune-Related Features and Immunotherapy Outcomes in Solid Tumors
Source: Front Immunol. 2022 Feb 23;13:799988. doi: 10.3389/fimmu.2022.799988 (PMC8905508; doi:10.3389/fimmu.2022.799988)

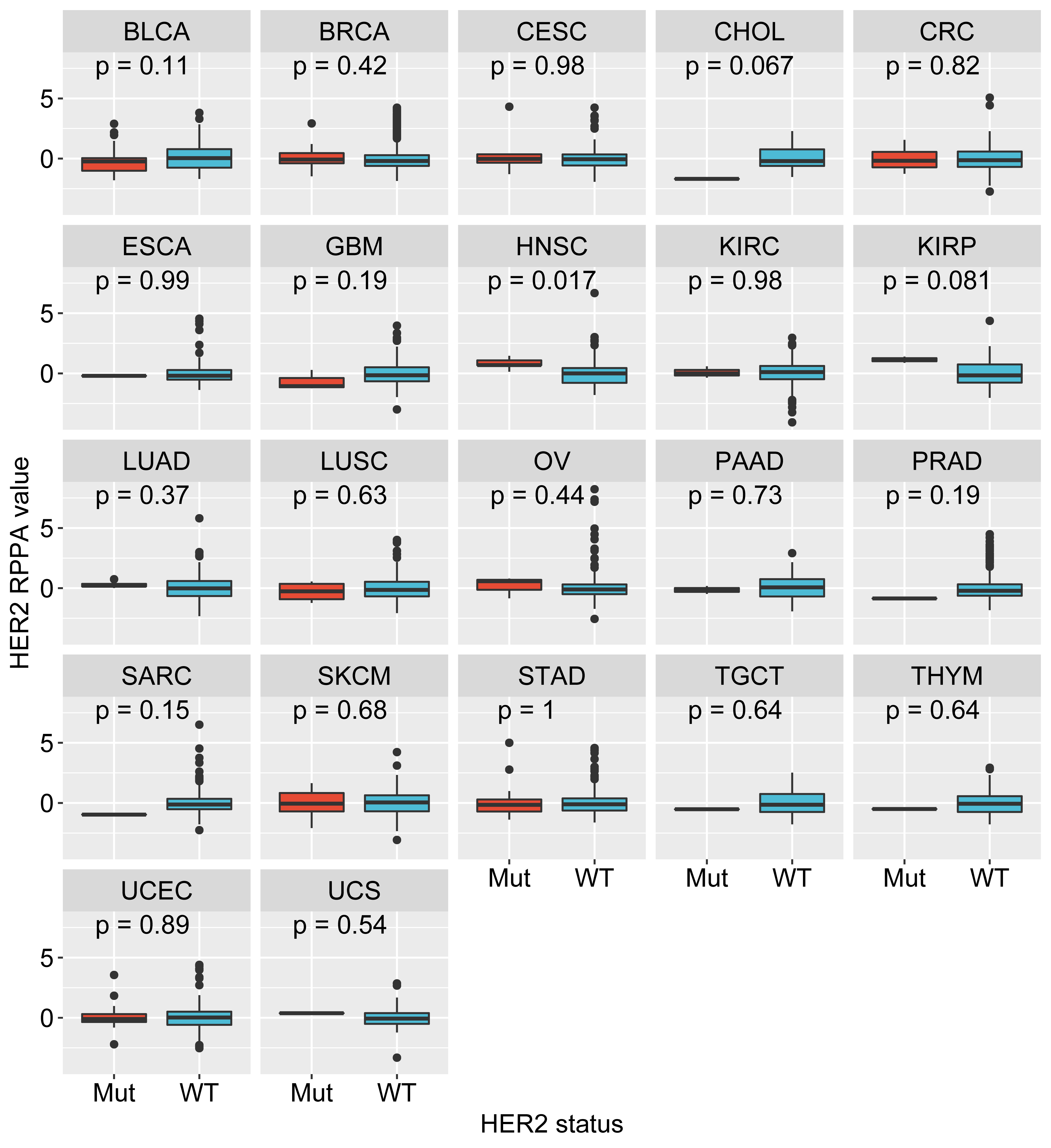

Supplement: Supplementary Figure S1 — Impact of HER2 mutation on HER2 protein expression by tumor types. RPPA, reverse-phase protein array; Mut, mutation; WT, wild-type. [file Image_1.tif]

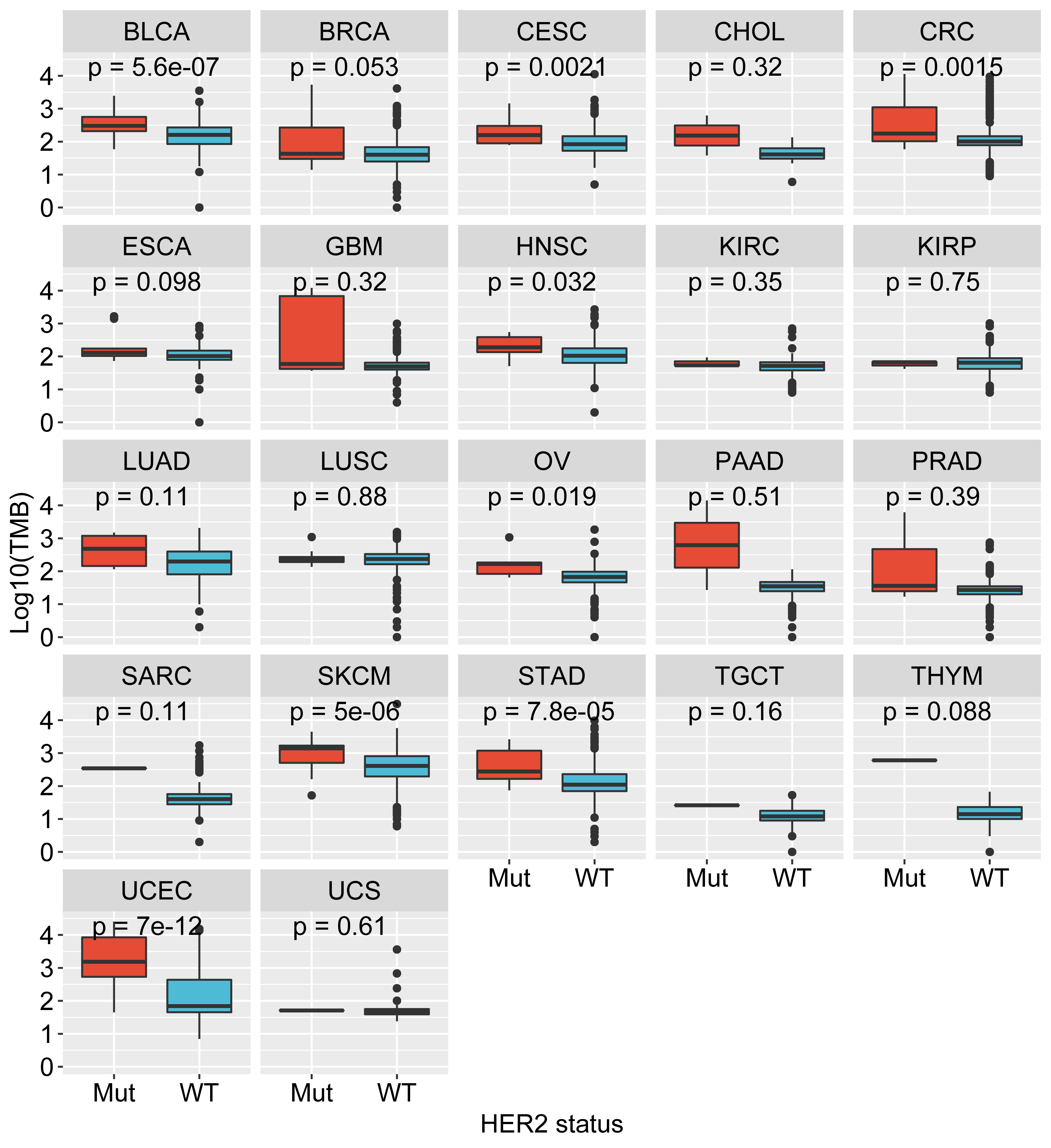

Supplement: Supplementary Figure S2 — Impact of HER2 mutation on tumor mutation burden (TMB) by tumor types. Mut, mutation; WT, wild-type. [file Image_2.tif]

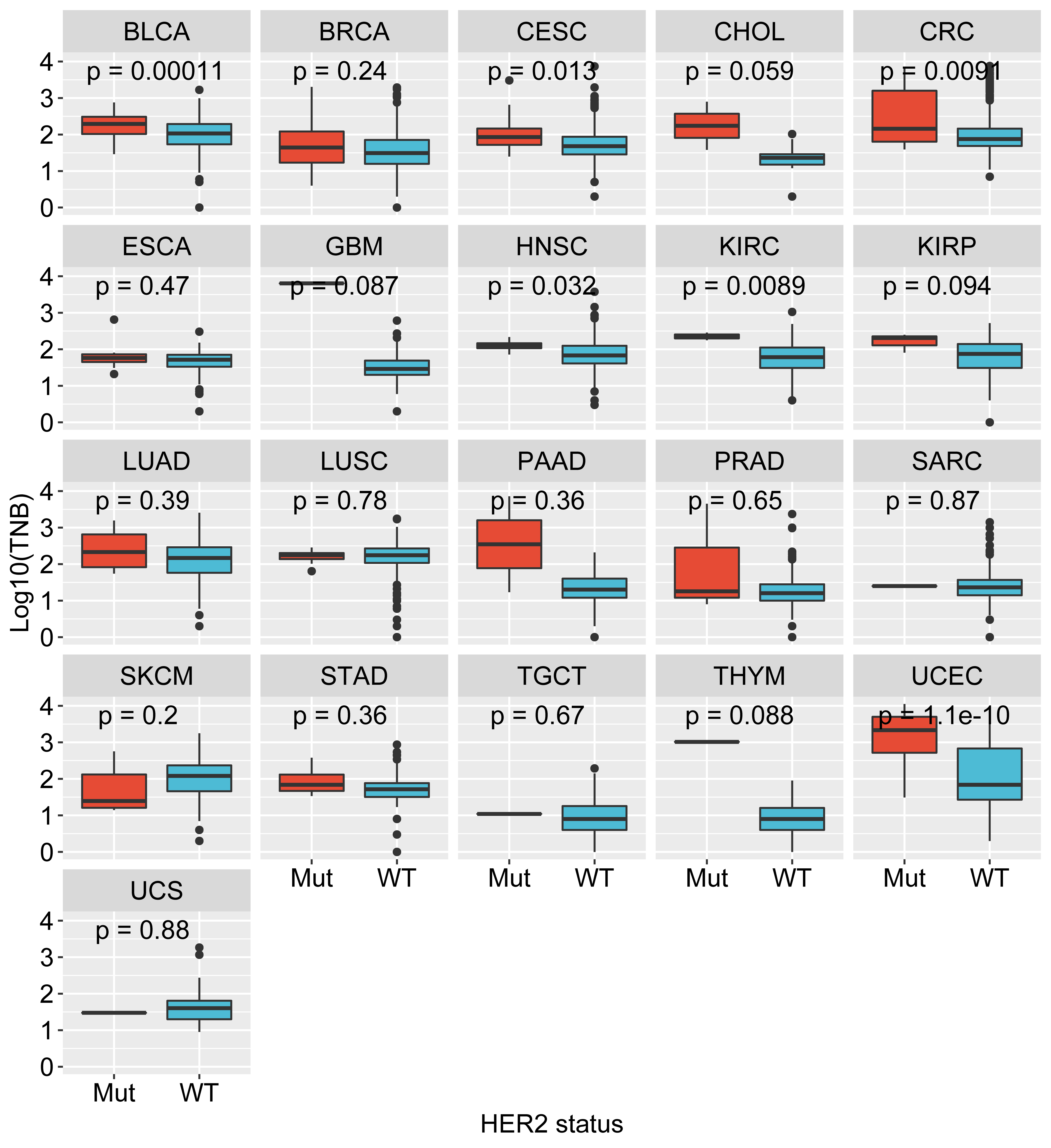

Supplement: Supplementary Figure S3 — Impact of HER2 mutation on tumor neoantigen burden (TNB) by tumor types; Mut, mutation; WT, wild-type. [file Image_3.tif]

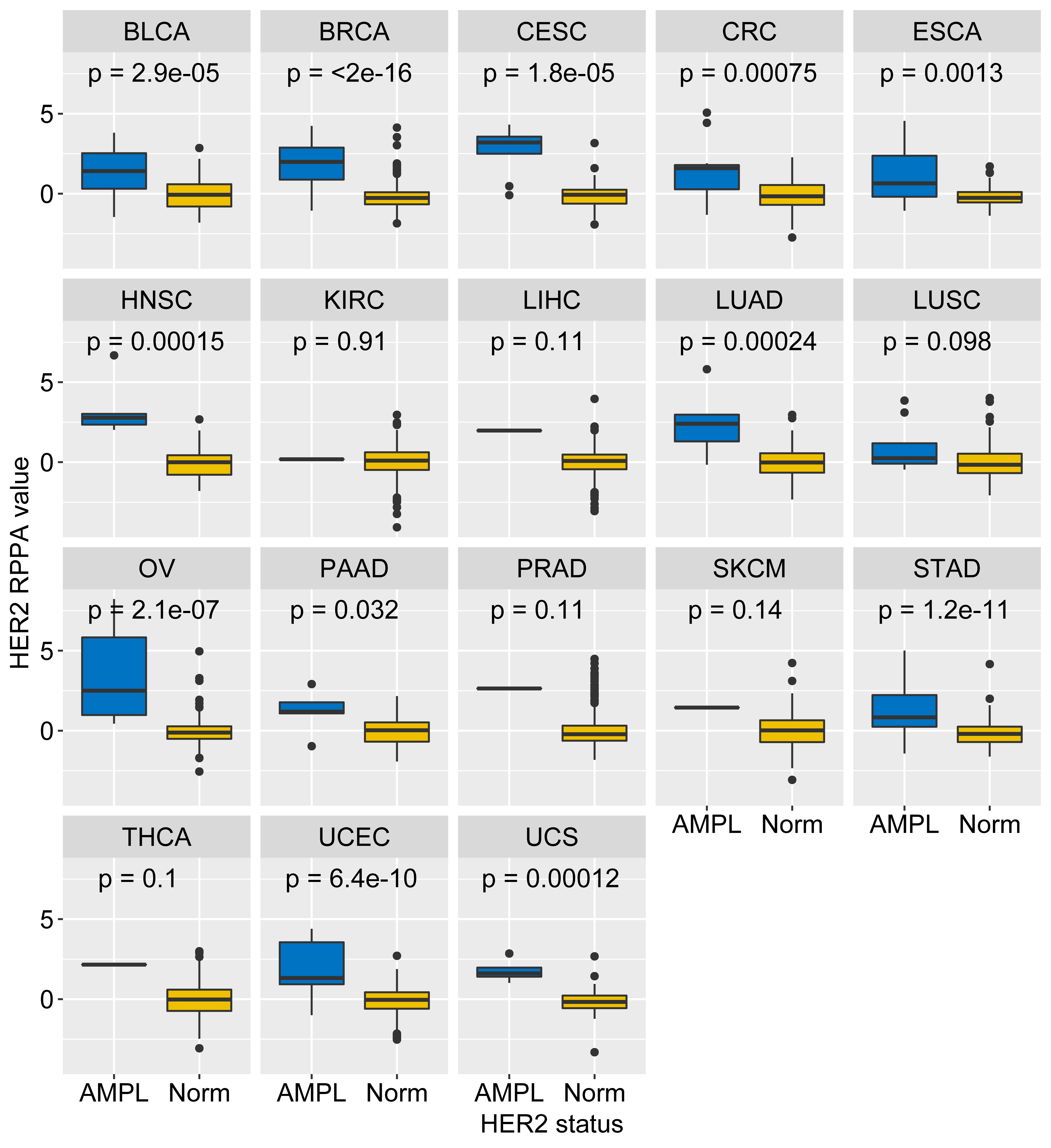

Supplement: Supplementary Figure S4 — Impact of HER2 amplification on HER2 protein expression by tumor types. RPPA, reverse-phase protein array; AMPL, amplification; Norm, normal. [file Image_4.tif]

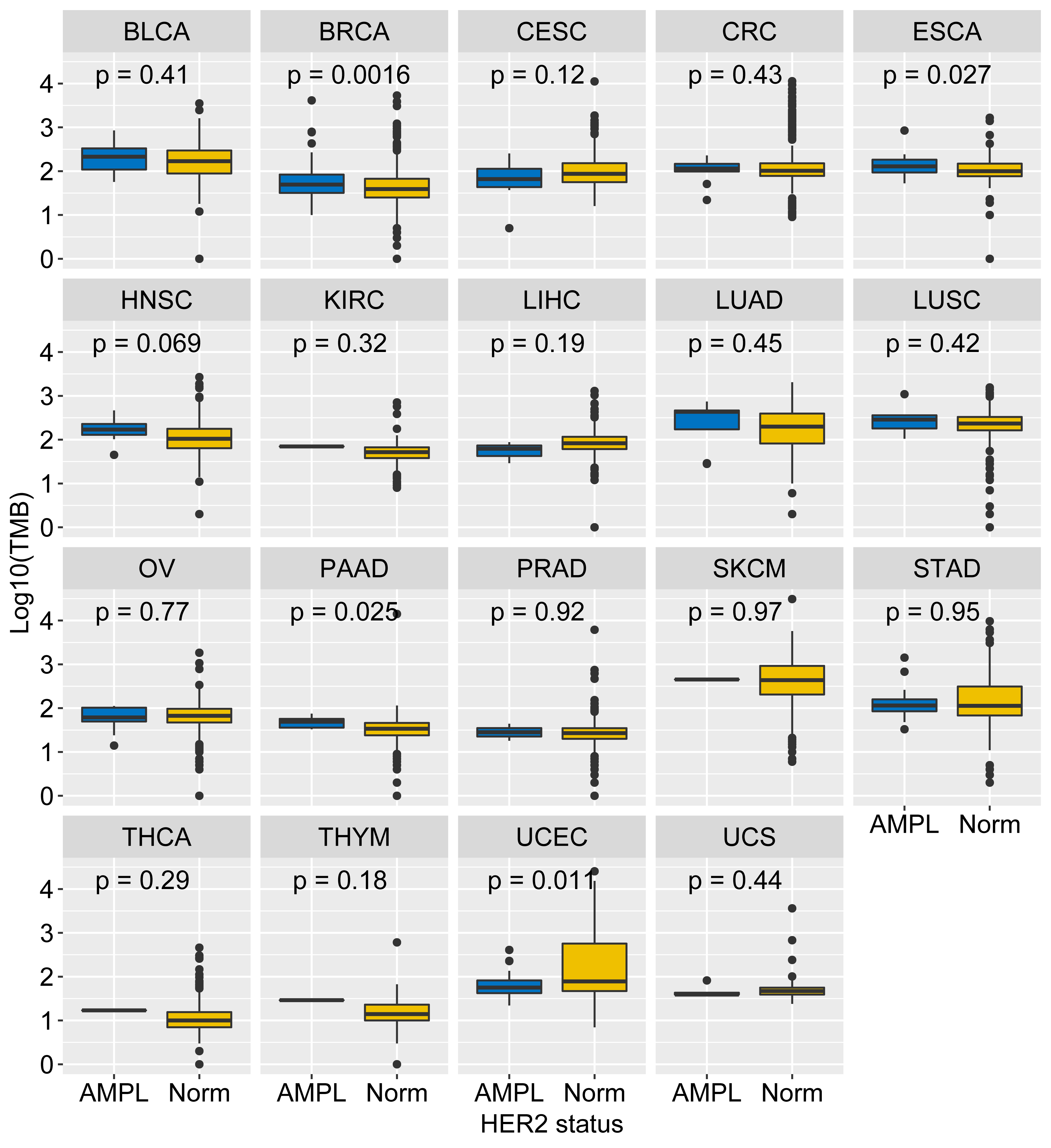

Supplement: Supplementary Figure S5 — Impact of HER2 amplification on tumor mutation burden (TMB) by tumor types. AMPL, amplification; Norm, normal. [file Image_5.tif]

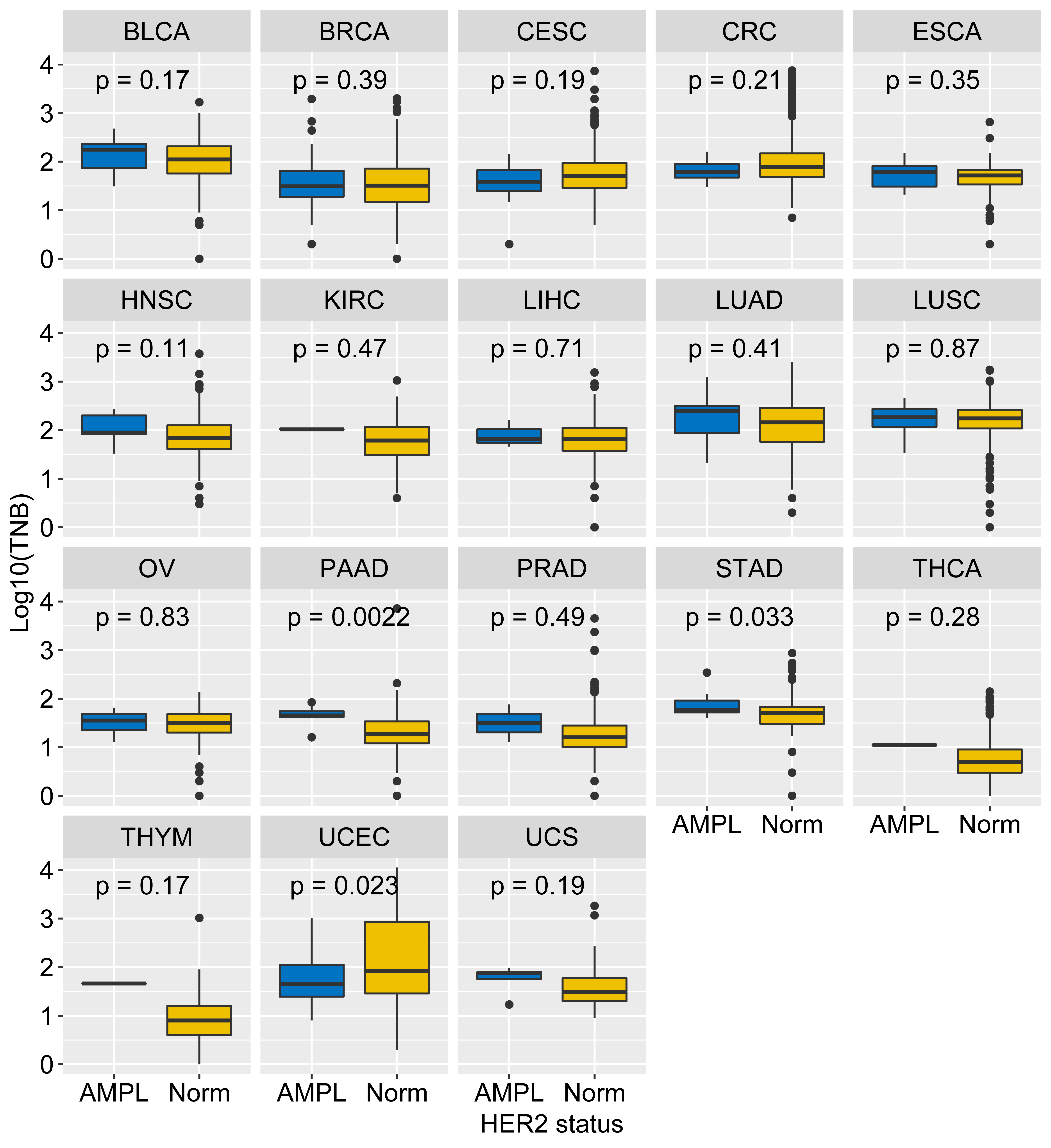

Supplement: Supplementary Figure S6 — Impact of HER2 amplification on tumor neoantigen burden (TNB) by tumor types. AMPL, amplification; Norm, normal. [file Image_6.tif]

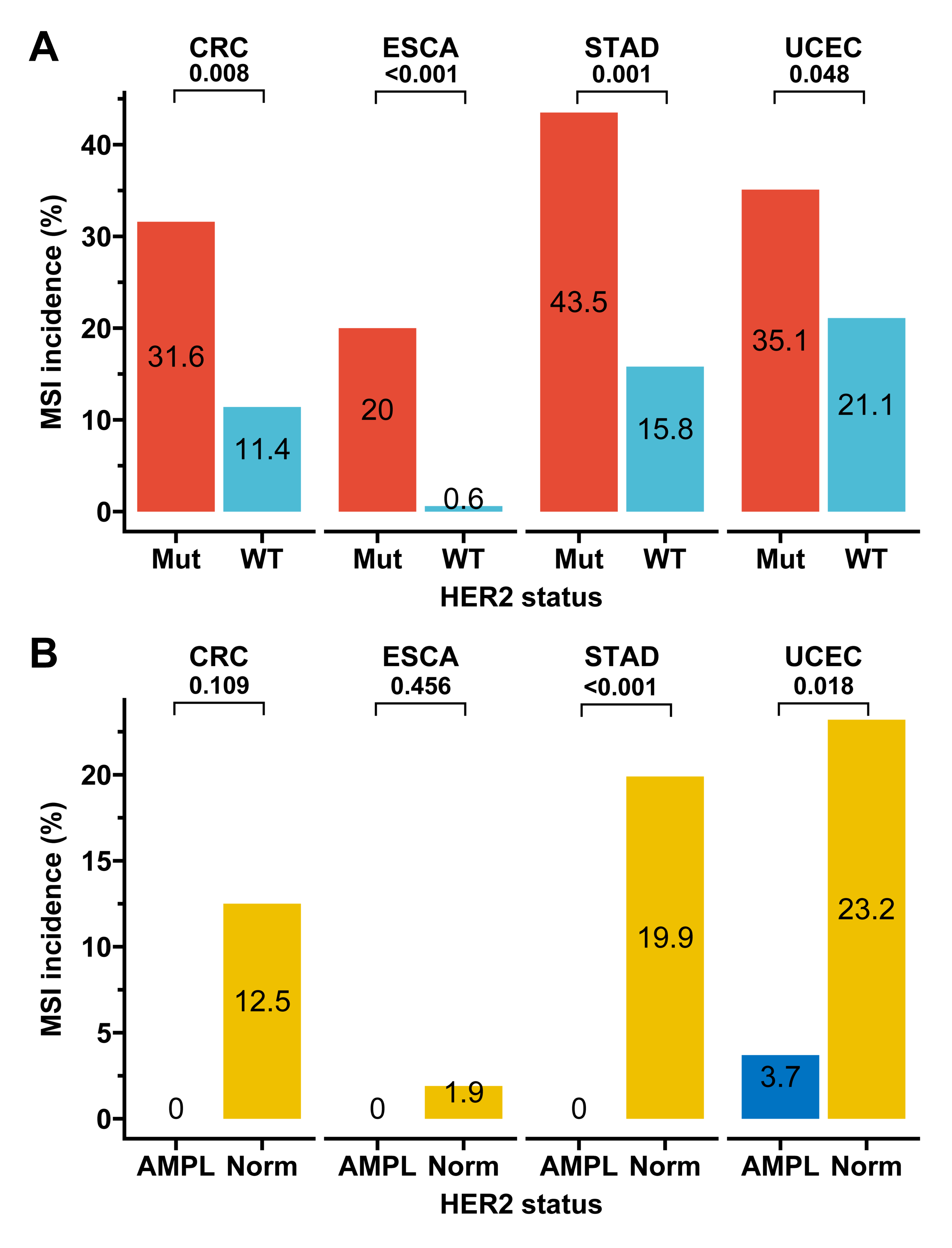

Supplement: Supplementary Figure S7 — Impact of HER2 alterations on microsatellite instability (MSI) by tumor types. (A, B) HER2 mutation (A) and amplification (B) impact MSI in 4 tumor types with a MSI incidence larger than 1%. Mut, mutation; WT, wild-type; AMPL, amplification; Norm, normal copy number. The MSI composition between subgroups was compared using the Chi-square or Fisher’s exact tests. [file Image_7.tif]

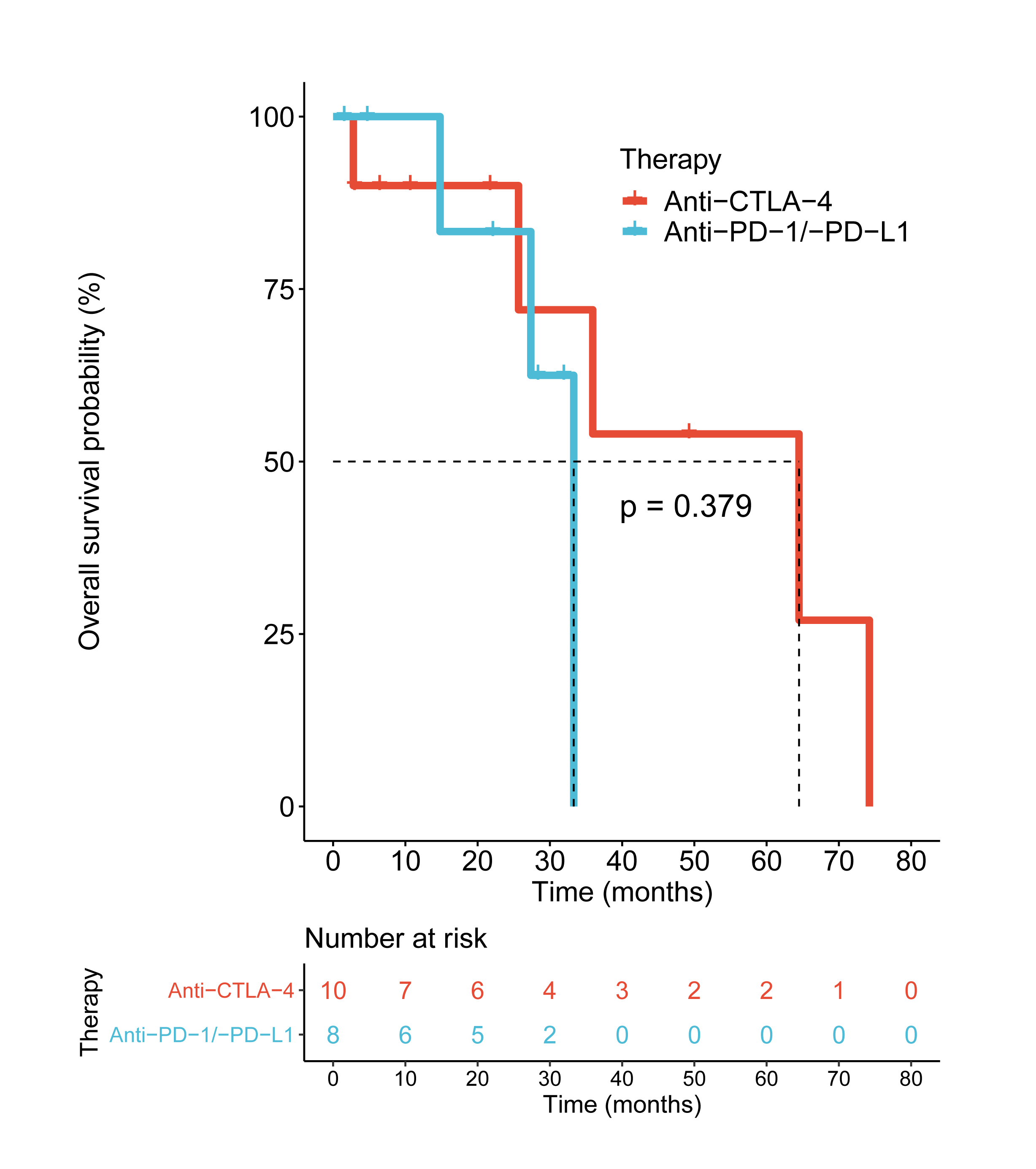

Supplement: Supplementary Figure S8 — Overall survival stratified by types of immune checkpoint inhibitors in the pooled immunotherapy cohort. [file Image_8.tif]
