# Supplementary material for: Associations of HER2 Mutation With Immune-Related Features and Immunotherapy Outcomes in Solid Tumors
Source: Front Immunol. 2022 Feb 23;13:799988. doi: 10.3389/fimmu.2022.799988 (PMC8905508; doi:10.3389/fimmu.2022.799988)
Supplement: Supplementary file 9 [file Table_1.docx]

**TABLE S1.** Microsatellite instability by HER2 mutation status in the TCGA pan-cancer cohort.

| **Tumor type^*^** | **Wild-type (%)** | **Mutation (%)** | **P value** |
| --- | --- | --- | --- |
| Colorectal cancer | | | |
| MSS | 452 (88.6) | 13 (68.4) | 0.008 |
| MSI | 58 (11.4) | 6 (31.6) |  |
| Esophageal carcinoma | | | |
| MSS | 171 (99.4) | 8 (80.0) | <0.001 |
| MSI | 1 (0.6) | 2 (20.0) |  |
| Stomach adenocarcinoma | | | |
| MSS | 347 (84.2) | 13 (56.5) | 0.001 |
| MSI | 65 (15.8) | 10 (43.5) |  |
| Uterine Corpus Endometrial Carcinoma | | | |
| MSS | 377 (78.9) | 24 (64.9) | 0.048 |
| MSI | 101 (21.1) | 13 (35.1) |  |

**^*^**Tumor types with an MSI incidence greater than 1%, and 1661 patients were selected.

TCGA: The Cancer Genome Atlas; MSI: microsatellite instability; MSS: microsatellite stability
