# Supplementary material for: Associations of HER2 Mutation With Immune-Related Features and Immunotherapy Outcomes in Solid Tumors
Source: Front Immunol. 2022 Feb 23;13:799988. doi: 10.3389/fimmu.2022.799988 (PMC8905508; doi:10.3389/fimmu.2022.799988)
Supplement: Supplementary file 10 [file Table_2.docx]

**TABLE S2.** Microsatellite instability by HER2 amplification status in the TCGA pan-cancer cohort.

| **Tumor type^*^** | **Norm (%)** | **AMPL (%)** | **P value** |
| --- | --- | --- | --- |
| Colorectal cancer | | | |
| MSS | 447 (87.5) | 18 (100.0) | 0.109 |
| MSI | 64 (12.5) | 0 (0.0) |  |
| Esophageal carcinoma | | | |
| MSS | 151 (98.1) | 28 (100.0) | 0.456 |
| MSI | 3 (1.9) | 0 (0.0) |  |
| Stomach adenocarcinoma | | | |
| MSS | 302 (80.1) | 58 (100.0) | <0.001 |
| MSI | 75 (19.9) | 0 (0.0) |  |
| Uterine Corpus Endometrial Carcinoma | | | |
| MSS | 375 (76.8) | 26 (96.3) | 0.015 |
| MSI | 113 (23.2) | 1 (3.7) |  |

**^*^**Tumor types with an MSI incidence greater than 1%, and 1661 patients were selected.

TCGA: The Cancer Genome Atlas; MSI: microsatellite instability; MSS: microsatellite stability; AMPL: amplification; Norm: normal copy number.
