# Supplementary material for: Associations of HER2 Mutation With Immune-Related Features and Immunotherapy Outcomes in Solid Tumors
Source: Front Immunol. 2022 Feb 23;13:799988. doi: 10.3389/fimmu.2022.799988 (PMC8905508; doi:10.3389/fimmu.2022.799988)
Supplement: Supplementary file 12 [file Table_4.docx]

**TABLE S4.** Therapy response by HER2 mutation status and tumor type in the pooled immunotherapy cohort.

| **Tumor type^*^** | **Wild-type (%)** | **Mutation (%)** | **P value** |
| --- | --- | --- | --- |
| Anal cancer | | | |
| Non-response | - | - | - |
| Response | - | 1 (100.0) |  |
| Bladder cancer | | | |
| Non-response | 13 (52.0) | 1 (50.0) | 0.957 |
| Response | 12 (48.0) | 1 (50.0) |  |
| Head and Neck squamous cell carcinoma | | | |
| Non-response | 9 (81.8) | 1 (100.0) | 0.640 |
| Response | 2 (18.2) | 0 (0.0) |  |
| Lung cancer | | | |
| Non-response | 40 (72.7) | 0 (0.0) | 0.027 |
| Response | 15 (27.3) | 2 (100.0) |  |
| Melanoma | | | |
| Non-response | 158 (77.1) | 8 (66.7) | 0.409 |
| Response | 47 (22.9) | 4 (33.3) |  |
